# Interface Cooperative Enhancement of PPY/Fe_2_O_3_@NF Composite Lithium Storage Material

**DOI:** 10.3390/polym18151875

**Published:** 2026-07-30

**Authors:** Lijun Zhang, Guojing Li, Xiaozhong Qi, Handi Xu, Huaqi Zhao, Meili Qi

**Affiliations:** School of Materials Science and Engineering, Jiamusi University, Jiamusi 154007, China

**Keywords:** α-Fe_2_O_3_, PPY, lithium-ion batteries

## Abstract

The evolution of electronic technology has intensified the demand for lithium-ion batteries to achieve elevated energy density, prolonged cycling longevity, and enhanced safety. Traditional graphite anodes are inadequate in meeting these requirements. α-Fe_2_O_3_ possesses a high theoretical capacity and is abundantly available; however, its poor conductivity and significant volume expansion during charge–discharge cycles restrict its practical applicability. This study addresses these issues by developing a three-layer cooperative structural anode material composed of foam nickel (NF), α-Fe_2_O_3_, and polypyrrole (PPY). Employing a hydrothermal method, α-Fe_2_O_3_ nanowires were in-situ grown on the 3D framework of foam nickel, followed by electro-polymerization to achieve a dense PPY coating. The foam nickel offers a highly conductive scaffold and mechanical support, while PPY enhances conductivity, provides structural buffering, and facilitates in-situ nitrogen doping. Collectively, these components synergistically improve conductivity, mitigate volume expansion, and optimize the solid electrolyte interphase. This composite material demonstrates comprehensive enhancements in conductivity, structural stability, and interface compatibility, as evidenced by the well-preserved structural integrity after prolonged cycling, thereby overcoming the performance limitations associated with singular α-Fe_2_O_3_ and simplistic composite systems, and presenting novel insights and technical support for the advancement of high-energy-density lithium-ion battery anodes.

## 1. Introduction

The rapid iteration of flexible electronic technologies demands lithium-ion batteries (LIBs) with higher energy density, extended cycle life, and enhanced safety [1,2]. However, conventional graphite anodes are fundamentally limited by their low theoretical specific capacity (372 mAh·g^−1^), which cannot satisfy the increasing energy density requirements for next-generation LIBs (e.g., 500 Wh·kg^−1^) [3]. Transition metal oxides (TMOs) have thus emerged as promising candidates [4]. Among them, α-Fe_2_O_3_ stands out due to its high theoretical capacity (1007 mAh·g^−1^), natural abundance, low cost, and environmental friendliness [5]. However, α-Fe_2_O_3_ suffers from two intrinsic drawbacks that drastically impede its practical application: poor electronic conductivity (10^−14^ to 10^−12^ S/cm) and severe volume expansion (up to ~200%) during lithiation/delithiation [6]. These issues cause electrode pulverization, loss of electrical contact, and continuous reformation of the solid electrolyte interphase (SEI), leading to rapid capacity decay and structural collapse [7].

To overcome these limitations, researchers have focused on designing unique nanostructures, incorporating conductive matrices (e.g., carbon nanotubes, MXenes), and developing rational electrode architectures [8]. In this regard, three-dimensional (3D) porous current collectors offer significant advantages. Nickel foam (NF), as a representative 3D scaffold, possesses high electronic conductivity (~10^4^ S/cm), a large specific surface area (50–500 m^2^/g), and excellent mechanical flexibility [9]. Crucially, compared to traditional 2D copper foil collectors, the robust 3D porous skeleton of NF can effectively accommodate the volume expansion of active materials, provides rapid electron transport pathways, and allows for the direct in-situ growth of active nanostructures [10]. Meanwhile, conductive polymers, particularly polypyrrole (PPY), are highly attractive due to their high conductivity, structural flexibility, and facile electropolymerization characteristics [11]. Importantly, the nitrogen atoms inherently present in the PPY molecules enable in-situ nitrogen doping, which increases electron density, creates additional redox-active sites for Li^+^ adsorption, and establishes efficient ion diffusion channels [12]. The flexible PPY shell can also act as a physical buffer to alleviate the mechanical stress of α-Fe_2_O_3_ during cycling and prevent direct electrode/electrolyte contact, thereby stabilizing the SEI film [13].

Despite the promising properties of individual α-Fe_2_O_3_/NF or PPY-coated systems, a systematic study integrating NF, α-Fe_2_O_3_, and PPY into a rationally designed tri-layer synergistic structure has been rarely reported [6,14]. Moreover, the synergistic coordination between the nitrogen species in PPY and the NiO native layers on the NF surface can further optimize the electrode/electrolyte interface [6,15]. Consequently, developing a straightforward and cost-effective strategy to construct such a “3D porous substrate-active core-conductive shell” architecture remains a core challenge [7,16].

Based on this, we propose a two-step synthesis strategy to fabricate a flexible PPY/α-Fe_2_O_3_@NF anode. First, α-Fe_2_O_3_ nanowires were in-situ grown on the 3D NF scaffold via a hydrothermal method to build a high-surface-area active core. Subsequently, a nitrogen-doped PPY layer was uniformly coated onto the α-Fe_2_O_3_ nanowires through mild electropolymerization. This trilayer design leverages the 3D NF framework for efficient electron transport and structural support, the α-Fe_2_O_3_ nanowires for high lithium storage, and the PPY coating for enhanced conductivity, volume buffering, and interfacial stabilization. As a result, the composite exhibits significantly improved electrochemical performance. In addition, our straightforward fabrication strategy is highly versatile and can be extended to other transition metal oxides (e.g., Co_3_O_4_, MnO_2_, NiO) for advanced lithium, sodium, and potassium-ion batteries, as well as flexible energy storage devices. This work provides fresh insights and an effective technical route for the design of high-energy-density flexible anodes.

## 2. Experimental Materials

The primary reagents and consumables utilized in this experiment are as follows: pyrrole (PPY) was procured from Shanghai Hongshun Biotechnology Co., Ltd. (Shanghai, China); anhydrous ethanol (C_2_H_5_OH, AR grade, purity 99.7%) and sulfuric acid (AR grade) were supplied by Chengdu Kegesi Biotechnology Co., Ltd. and Chengdu Kelong Chemical Co., Ltd. (Chengdu, China), respectively; polyvinylidene fluoride (PVDF, molecular weight 1,000,000) was obtained from Dongguan Kede Innovation Technology Co., Ltd. (Dongguan, China); the electrolyte MJS-lbe01-180101 was sourced from Nanjing MJS Energy Technology Co., Ltd. (Nanjing, China); CR2032 battery casings were purchased from Taizhou Yajun Battery Material Co., Ltd. (Taizhou, China); lithium foil (purity > 99%) was provided by Xinghua Benoit (Xinghua, China); nickel foam (2,001,001.5 mm, 2 pcs, ppi: 110) was supplied by Kunshan Dongzhimei Electronics Co., Ltd. (Kunshan, China); microporous polyethylene separators (Celgard 2320), and electrochemical testing-related materials such as vinylene carbonate/dimethyl carbonate/methyl carbonate (volume ratio 1:1:1) was procured Jinfuchen New Materials Technology Co., Ltd. (Guangdong, China).

### 2.1. Electrode Preparation

#### Synthesis of α-Fe_2_O_3_@NF Electrode

The α-Fe_2_O_3_@NF nanowire array was synthesized via a hydrothermal method, with the following specific steps: Dissolve 0.24 g of Na_2_SO_4_·6H_2_O and 0.47 g of FeCl_3_·6H_2_O in 35 mL of deionized water, stirring until a homogeneous hydrothermal reaction solution is formed; place the reaction solution together with the nickel foam (NF) into a steel-lined polytetrafluoroethylene reaction vessel and subject it to a constant temperature of 160 °C for 6 h; after natural cooling, rinse the product five times alternately with anhydrous ethanol and deionized water, and then dry in an oven at 50 °C to obtain the α-Fe_2_O_3_@NF precursor; subsequently, place the precursor in a tube furnace and heat to 450 °C at a rate of 10 °C·min^−1^, maintaining for 2 h, then cool to room temperature to acquire the α-Fe_2_O_3_@NF electrode.

### 2.2. Preparation of α-Fe_2_O_3_/PPY@NF-30 Electrode

**Figure 1:**
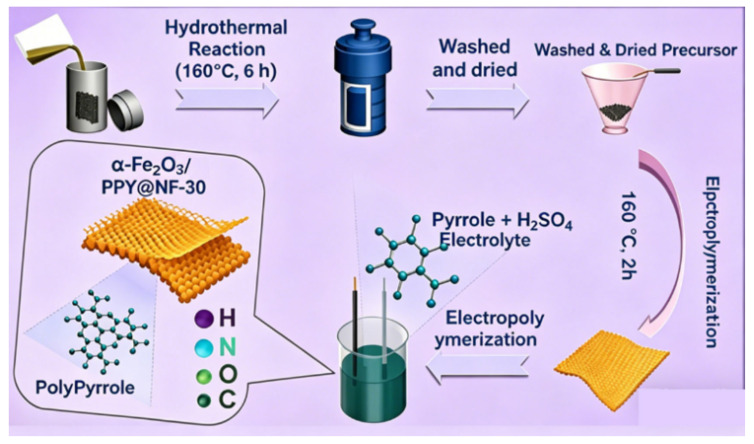
Schematic illustration of the fabrication procedure for α-Fe_2_O_3_/PPY@NF-30.

The α-Fe_2_O_3_/PPY@NF-30 electrode was fabricated via a two-step electropolymerization method (as illustrated in Figure 1). First, the electrodeposition solution was prepared by slowly adding 1 mL of concentrated sulfuric acid into 100 mL of deionized water under continuous stirring. Subsequently, 1 mL of pyrrole monomer was added, and the mixture was stirred until a uniform and homogeneous solution was obtained. Second, a piece of α-Fe_2_O_3_@NF (0.8 × 0.8 cm) was used as the working electrode (anode), with a high-purity graphite rod as the counter electrode (cathode). The electropolymerization was performed in the above solution using a galvanostatic anodic deposition mode with a constant current of 0.03 A for 30 s. After the deposition, the resulting product was washed alternately with anhydrous ethanol and deionized water and then dried to obtain the final α-Fe_2_O_3_/PPY@NF-30 electrode.

### 2.3. Structural and Morphological Characterization

Various characterization techniques were employed to analyze the structure, morphology, and elemental composition of the electrodes: Morphological characterization was performed using field emission scanning electron microscopy (FE-SEM, JEOL JSM-7800F, JEOL Ltd., Tokyo, Japan) to observe the surface morphology of the electrodes; the microscopic structure was analyzed via transmission electron microscopy (TEM, Hitachi HT7700, Hitachi High-Tech, Tokyo, Japan); elemental distribution was characterized using energy-dispersive X-ray spectroscopy (EDS, Oxford Instruments, Abingdon, UK) to assess surface elemental distribution; the crystal structure was examined using X-ray diffraction (XRD, Bruker D8 Advance, Bruker AXS GmbH, Karlsruhe, Germany) with Cu-Kα radiation (λ = 1.5406 Å); chemical structure was investigated through Fourier-transform infrared spectroscopy (FT-IR, Bruker Tensor 270, Bruker Optics, Ettlingen, Germany) to study the chemical bonding characteristics of the electrodes; Raman spectra were recorded using a Renishaw InVia confocal Raman spectrometer (Renishaw plc, Wotton-under-Edge, UK) with a 5 mW argon ion laser (excitation wavelength 514.5 nm, under room temperature conditions); and elemental binding energies were analyzed through X-ray photoelectron spectroscopy (XPS, ESCALAB MK II, VG Scientific/Thermo Fisher Scientific, East Grinstead, UK) equipped with a monochromatic X-ray source.

### 2.4. Electrochemical Performance Testing

#### Battery Assembly

The CR2032 coin cell was utilized as the testing system, assembled within an argon-filled glovebox: Both α-Fe_2_O_3_@NF and α-Fe_2_O_3_/PPY@NF-30 electrodes were cut into 0.8 × 0.8 cm pieces to serve as working electrodes; lithium foil was employed as the counter/reference electrode, and Celgard 2320 served as the separator; the electrolyte comprised 80 μL of a 1.0 M LiPF_6_ mixed solution of vinylene carbonate/dimethyl carbonate/methyl carbonate (in a volume ratio of 1:1:1).

### 2.5. Testing Methods

Galvanostatic charge–discharge testing (GCD) was conducted using a LAND battery testing system (CT2001A, Wuhan LAND Electronic Co., Ltd., Wuhan, China) across a voltage range of 0.1 to 3.0 V to evaluate the charge–discharge performance of the electrodes; cyclic voltammetry (CV) was performed utilizing a Shanghai Chenhua electrochemical workstation (CHI660E, Shanghai Chenhua Instrument Co., Ltd., Shanghai, China) at a scan rate of 0.1 mV·s^−1^ within a voltage range of 0.1 to 3.0 V to analyze the redox behavior of the electrodes; electrochemical impedance spectroscopy (EIS) was conducted through a PARSTAT electrochemical workstation (P4000A, Princeton Applied Research (PAR), Oak Ridge, TN, USA) to investigate the battery impedance characteristics over a frequency range of 10^−3^ to 10^4^ Hz; all electrochemical tests were carried out under ambient temperature conditions.

## 3. Results and Discussion

### 3.1. Electrode Characterization

**Figure 2:**
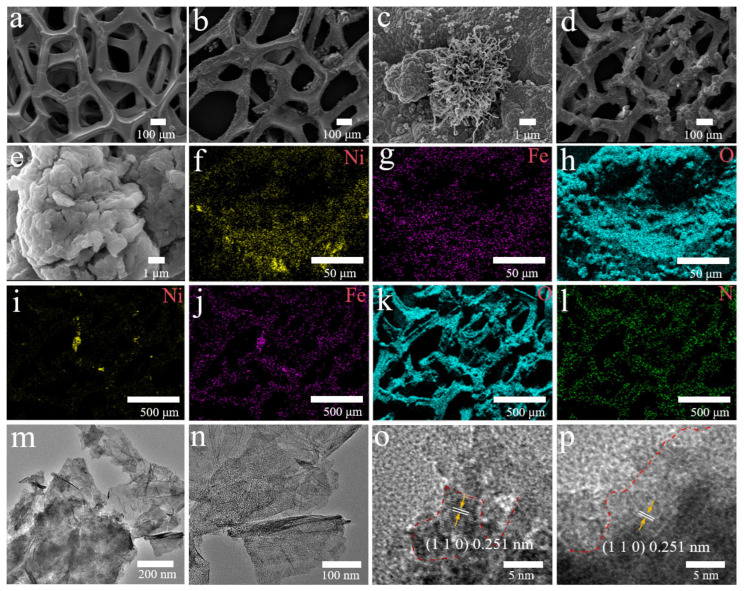
Presents the field-emission scanning electron microscopy (FESEM), energy-dispersive spectroscopy (EDS), and transmission electron microscopy (TEM) characterizations of NF, α-Fe_2_O_3_@NF, and α-Fe_2_O_3_/PPY@NF-30. Specifically, (**a**) shows the FESEM image of NF; (**b**,**c**) display the FESEM images of α-Fe_2_O_3_@NF; (**d**,**e**) illustrate the FESEM images of α-Fe_2_O_3_/PPY@NF-30; (**f**–**h**) depict the EDS elemental mappings of nickel, iron, and oxygen in α-Fe_2_O_3_@NF (Figure S1), (**i**–**l**) show the EDS elemental mappings of nickel, iron, oxygen, and nitrogen in α-Fe_2_O_3_/PPY@NF-30 (Figure S2); (**m**,**n**) are TEM images of α-Fe_2_O_3_/PPY; (**o**,**p**) are high-resolution TEM (HRTEM) images of α-Fe_2_O_3_/PPY, where α-Fe_2_O_3_/PPY was obtained by exfoliating from α-Fe_2_O_3_/PPY@NF-30 (The arrows in the figure represent the measured lattice spacing, and the red dotted line is the boundary line between the two substances).

Figure 2a–e present the FE-SEM images of NF, α-Fe_2_O_3_@NF, and α-Fe_2_O_3_/PPY@NF-30, respectively. As observed in Figure 2b,c, α-Fe_2_O_3_ particles were successfully deposited on the NF surface via hydrothermal synthesis. Subsequently, a uniform PPY coating was formed on the α-Fe_2_O_3_@NF surface through electropolymerization with a deposition time of 30 s (Figure 2d,e). Morphological analysis reveals that the α-Fe_2_O_3_/PPY@NF-30 electrode possesses a distinct core–shell structure with improved adhesion of the electrode material [3,17]. This dense and homogeneous encapsulation effectively enlarges the electrochemically active contact area between the electrolyte and the active material, thereby facilitating improved reaction kinetics.

The dense and homogeneous encapsulation structure also contributes to enhanced cycling stability of the α-Fe_2_O_3_/PPY@NF-30 electrode. The elemental mapping results (Figure 2f–l) of α-Fe_2_O_3_@NF and α-Fe_2_O_3_/PPY@NF-30 confirm the uniform distribution of both α-Fe_2_O_3_ and PPY on the NF scaffold, further verifying the successful fabrication of the composite. This aligns with the electrode morphology observations, demonstrating the homogeneous distribution of Fe, O, and N elements on the electrode surface (additional FE-SEM images are provided in the Supporting Information, as shown in Figures S1 and S2). Figure 2m–p display TEM images of the α-Fe_2_O_3_/PPY electrode [4]. The interplanar spacing of the (110) crystal plane of α-Fe_2_O_3_ is measured as 0.251 nm (2.51 Å), consistent with standard α-Fe_2_O_3_. Concurrently, the core–shell structure of α-Fe_2_O_3_/PPY is clearly visible, confirming that the prepared sample exhibits a core–shell configuration of α-Fe_2_O_3_/PPY@NF-30, with the α-Fe_2_O_3_/PPY obtained by exfoliation from the α-Fe_2_O_3_/PPY@NF-30 sample.

**Figure 3:**
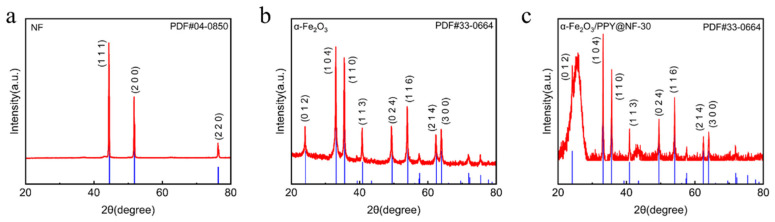
(**a**–**c**) depict the XRD patterns of NF, α-Fe_2_O_3_@NF, and α-Fe_2_O_3_/PPY@NF-30, respectively (The red line in the figure represents the result data, while the blue line is the standard comparison card).

The XRD patterns of the resultant NF, α-Fe_2_O_3_@NF, and α-Fe_2_O_3_/PPY@NF-30 electrodes are presented in Figure 3a–c. The XRD pattern of α-Fe_2_O_3_ is consistent with that of hematite α-Fe_2_O_3_ (PDF#33-0664). Both α-Fe_2_O_3_@NF and α-Fe_2_O_3_/PPY@NF-30 electrodes exhibit characteristic diffraction peaks corresponding to hematite α-Fe_2_O_3_, with no discernible impurity peaks, indicating the formation of high-purity α-Fe_2_O_3_. Notably, the crystallinity of α-Fe_2_O_3_ remains unaffected by the PPY coating [5]. However, a close examination of the comparative patterns reveals that while the (012) diffraction peak of the pristine α-Fe_2_O_3_ remains unchanged, an additional broad peak appears at a slightly lower angle (higher d-spacing) adjacent to the (012) reflection in the α-Fe_2_O_3_/PPY@NF-30 sample. This additional feature can be attributed to the strong interfacial interaction between the PPY coating and the α-Fe_2_O_3_ lattice, which may induce localized structural distortion or the overlap of the semi-crystalline PPY phase, rather than a global loss of crystallinity.

**Figure 4:**
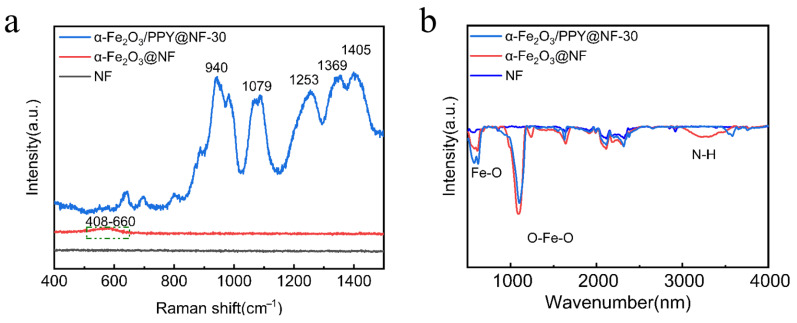
(**a**) illustrates the Raman spectra of NF, α-Fe_2_O_3_@NF, and α-Fe_2_O_3_/PPY@NF-30, while (**b**) presents their corresponding Fourier-transform infrared (FTIR) spectra.

Raman spectroscopy was employed to further verify the composition of the electrodes (Figure 4a). The spectral bands of α-Fe_2_O_3_ exhibit vibrations corresponding to Fe-O bonds within the range of 408–660 cm^−1^. The presence of D and G band peaks in the spectrum of α-Fe_2_O_3_/PPY@NF-30 confirms the existence of defects in the carbon framework. These defects can provide additional active sites for Li^+^ storage, thereby enhancing the electrochemical performance of the electrode. The D band is attributed to disordered carbon, while the G band corresponds to graphitic carbon. The relative intensity of these bands indicates the extent of defects, which is crucial for facilitating Li+ storage by generating more reactive sites. In addition to the D and G bands, characteristic Raman peaks at 940, 1079, 1253, 1369, and 1405 cm^−1^ are clearly observed in the α-Fe_2_O_3_/PPY@NF-30 spectrum, which are well-indexed to the characteristic vibrational modes of the PPY polymer backbone. The aforementioned analysis is consistent with the XRD results. The FT-IR spectra of NF, α-Fe_2_O_3_@NF, and α-Fe_2_O_3_/PPY@NF-30 are comparatively presented in Figure 4b. Both α-Fe_2_O_3_@NF and α-Fe_2_O_3_/PPY@NF-30 electrodes exhibit an absorption band at approximately 538 cm^−1^, corresponding to the stretching vibration of the Fe-O functional group. Notably, a prominent characteristic absorption peak at 3347 cm^−1^ is observed in the α-Fe_2_O_3_/PPY@NF-30 electrode, attributed to the N-H stretching vibration of PPY. Based on these results, the successful synthesis of α-Fe_2_O_3_/PPY@NF-30 is confirmed [9,10].

**Figure 5:**
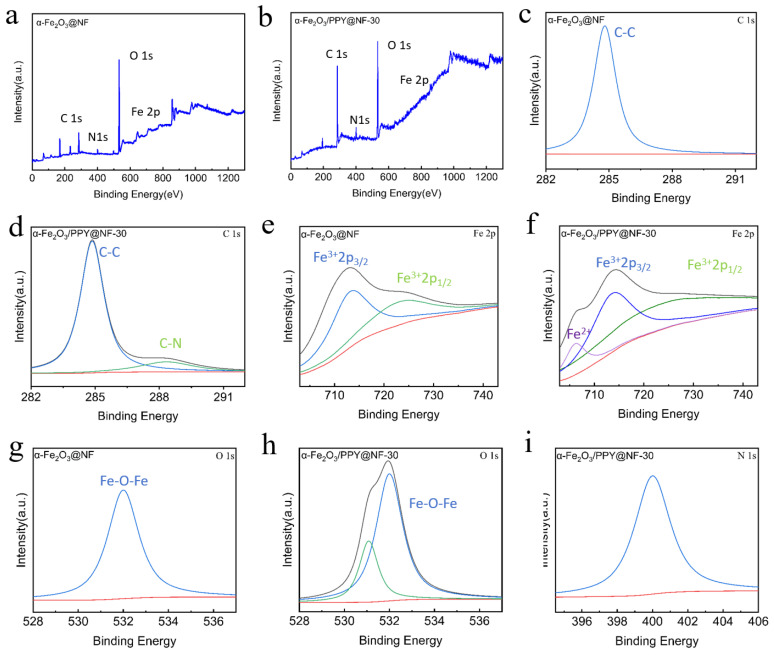
Presents the XPS spectra of α-Fe_2_O_3_@NF and α-Fe_2_O_3_/PPY@NF-30, including survey spectra (**a**,**b**), C 1s spectra (**c**,**d**), Fe 2p spectra (**e**,**f**), O 1s spectra (**g**,**h**), and N 1s spectrum (**i**) for α-Fe_2_O_3_/PPY@NF-30 (The grey lines represent the data, while the red lines represent the background of the peak fitting).

XPS analyses were performed to elucidate ligand formation, coordination bonds, and the valence states of iron ions, with the results depicted in Figure 5. Characteristic peaks corresponding to C, Fe, N, and O are evident in the XPS survey spectra (Figure 5a,b), and the compositional data for α-Fe_2_O_3_/PPY@NF-30 are in agreement with the EDS results. In the high-resolution C 1s spectra (Figure 5c,d), peaks at 284.6 and 288.8 eV are assigned to C–C and C–N functional groups, respectively; the former arises primarily from adventitious carbon, while the latter originates from the pyrrole ring backbone. The Fe 2p spectra (Figure 5e,f) are deconvoluted into two spin–orbit doublets, with peaks at 724.6 and 711.4 eV attributed to Fe^3+^ and a peak at 708.3 eV assigned to Fe^2+^, indicating partial reduction in Fe^3+^ to Fe^2+^ in α-Fe_2_O_3_/PPY@NF-30. The O 1s spectra (Figure 5g,h) confirm the presence of Fe–O–Fe and Fe–O bonds, with component peaks at 532.7 and 529.78 eV, respectively, the latter being characteristic of the iron oxide. The N 1s spectrum of α-Fe_2_O_3_/PPY@NF-30 (Figure 5i) exhibits a peak at 399.9 eV, which is assigned to pyrrolic nitrogen. The intrinsic pyrrolic nitrogen in the polypyrrole matrix enhances Li^+^ adsorption capacity by providing abundant electroactive sites and creates effective pathways for Li^+^ diffusion. The intrinsic nitrogen in the polypyrrole matrix enhances adsorption capacity by furnishing additional redox-active sites and promotes Li^+^ diffusion. Consequently, nitrogen-doped α-Fe_2_O_3_ demonstrates markedly improved electrochemical performance, particularly in ion transport, owing to the increased density of surface active sites that facilitate lithium-ion interactions [3,6]. Collectively, the XPS results substantiate the successful fabrication of the α-Fe_2_O_3_/PPY@NF-30 architecture, which is consistent with the FTIR spectroscopic findings.

### 3.2. Electrochemical Characterization

**Figure 6:**
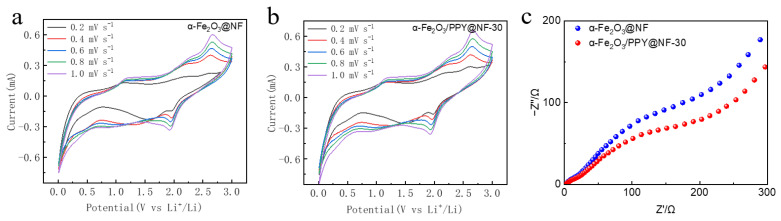
Presents the cyclic voltammetry (CV) curves of (**a**) α-Fe_2_O_3_@NF and (**b**) α-Fe_2_O_3_/PPY@NF-30 electrodes at scan rates ranging from 0.2 to 1 mV s^−1^, as well as (**c**) a comparative Nyquist plot for α-Fe_2_O_3_@NF and α-Fe_2_O_3_/PPY@NF-30.

Cyclic voltammetry (CV) measurements were conducted to further investigate the lithium storage capabilities of the electrodes (Figure 6). Figure 6a,b present the voltammetric profiles of the α-Fe_2_O_3_@NF and α-Fe_2_O_3_/PPY@NF-30 electrodes at scan rates ranging from 0.2 to 1.0 mV s^−1^. Both electrodes exhibit well-defined redox peaks throughout the cycling process. During the initial cathodic scan, the observed cathodic peak is attributable to the reduction process. This phenomenon is also associated with Li^+^ insertion into the Fe_2_O_3_ lattice and the formation of a solid electrolyte interphase (SEI) layer derived from electrolyte decomposition, which contributes to a lower initial Coulombic efficiency. Quantitative integration of the CV curves reveals a noticeably larger enclosed area for α-Fe_2_O_3_/PPY@NF-30, indicating higher specific capacity and enhanced charge storage capability. Additionally, the sharper and more symmetric redox peaks suggest enhanced electrochemical reversibility and improved reaction kinetics after PPY coating. Based on the established lithium storage mechanism of Fe_2_O_3_ and the aforementioned results, the plausible electrochemical reactions are proposed as follows:

(1) $${Fe}_{2}O_{3}+6Li+6e^{-}↔{2Fe}^{0}+{3Li}_{2}O$$

Figure 6c displays the Nyquist plots of the α-Fe_2_O_3_@NF and α-Fe_2_O_3_/PPY@NF-30 electrodes after 100 cycles at a current density of 100 mA g^−1^, measured over a frequency range from 0.01 to 100,000 Hz. Each Nyquist plot comprises a semicircle in the high-frequency region and a diagonal line in the low-frequency region, corresponding to the Warburg impedance [18]. The intercept at the highest frequency on the real axis, denoted as R_s_, represents the ohmic resistance associated with Li^+^ transport in the electrolyte [19,20]. The diameter of the semicircle in the high-frequency region indicates the interfacial charge transfer resistance (Rct), which is related to the Li^+^ ion transference at the interface between the active material and the electrolyte. Concurrently, the sloping line in the low-frequency region is associated with Li^+^ diffusion within the active material [21]. An equivalent circuit model was established to fit the EIS data (as shown in the illustration in Figure 6c). The fitting results reveal that the α-Fe_2_O_3_/PPY@NF-30 electrode exhibits a significantly reduced Rct compared to α-Fe_2_O_3_@NF, confirming that the PPY coating effectively lowers the charge-transfer barrier [22]. Moreover, the integration of the core–shell PPY structure with the α-Fe_2_O_3_@NF scaffold establishes a conductive network, substantially improving both ionic and electronic conductivity. In the high-frequency region, the Rct of the α-Fe_2_O_3_/PPY@NF-30 electrode is markedly lower than that of α-Fe_2_O_3_@NF, indicating reduced hindrance to Li^+^ transfer across the electrode/electrolyte interface. This improvement is attributed to the introduction of PPY, which facilitates the formation of a stable SEI layer at the interface, thereby enhancing lithium-ion transfer efficiency.

**Figure 7:**
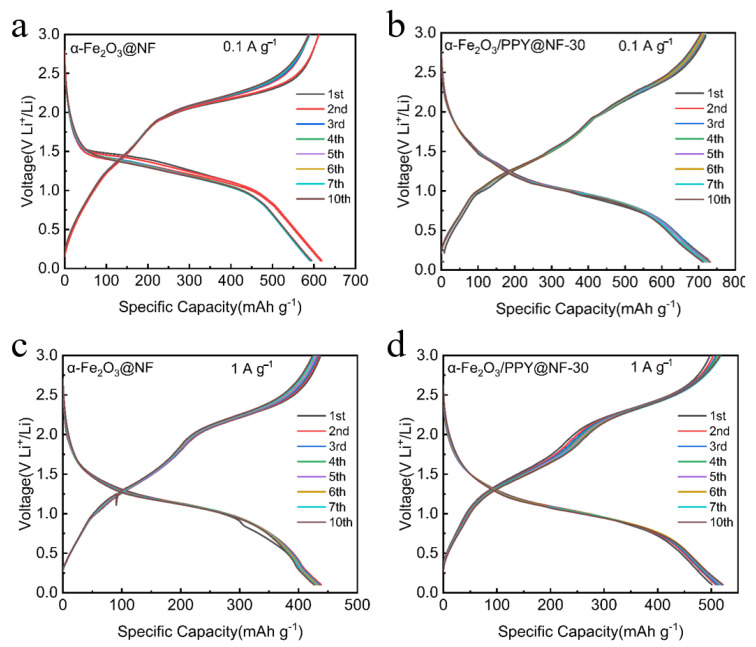
Presents the galvanostatic charge–discharge voltage profiles of (**a**) α-Fe_2_O_3_@NF and (**b**) α-Fe_2_O_3_/PPY@NF-30 at a current density of 0.1 mA g^−1^, as well as (**c**) α-Fe_2_O_3_@NF and (**d**) α-Fe_2_O_3_/PPY@NF-30 at a current density of 1 A g^−1^.

The galvanostatic charge–discharge profiles of α-Fe_2_O_3_@NF and α-Fe_2_O_3_/PPY@NF-30 electrodes at 0.1 A g^−1^ are depicted in Figure 7a,b, respectively. The discharge voltage plateau at approximately 0.81 V is associated with the reduction in Fe^3+^ to Fe^0^, whereas the charge voltage plateau at around 1.31 V corresponds to the delithiation of LixFe_2_O_3_. As illustrated in Figure 7a,b, the α-Fe_2_O_3_/PPY@NF-30 electrode delivers an initial discharge capacity of 741 mAh g^−1^, significantly surpassing that of α-Fe_2_O_3_@NF (618 mAh g^−1^). After 10 cycles, the α-Fe_2_O_3_/PPY@NF-30 electrode retains a capacity of 721 mAh g^−1^, while α-Fe_2_O_3_@NF exhibits a capacity of 598 mAh g^−1^. The enhanced capacity is primarily attributed to the synergistic effect of the PPY coating, which not only forms a compact encapsulation layer to provide structural protection, but also introduces nitrogen-doped active sites for lithium-ion storage, as confirmed by the XPS and Raman analyses [23,24]. The observed irreversible capacity loss during the initial cycles is mainly due to the formation of the SEI layer or electrolyte decomposition, as corroborated by the CV curves. The α-Fe_2_O_3_/PPY@NF-30 electrode exhibits excellent reversibility, with minimal capacity fading upon prolonged cycling. The nitrogen-containing PPY provides expedited ion transport channels, enhances the electrochemical conversion efficiency during lithiation/delithiation, and prevents direct contact between the electrode and electrolyte, thereby effectively buffering volume expansion and improving cycling stability. Furthermore, the introduction of the PPY coating mitigates electrolyte decomposition and irreversible lithium-ion intercalation, effectively augmenting the reversible capacity. Figure 7c,d display the galvanostatic charge–discharge profiles of α-Fe_2_O_3_@NF and α-Fe_2_O_3_/PPY@NF-30 electrodes at 1 A g^−1^, respectively. Notably, the α-Fe_2_O_3_/PPY@NF-30 electrode maintains an outstanding rate capability (505 mAh g^−1^) under high current densities [25,26]. The electrodeposited coordination facilitates controlled accumulation of the conductive polymer, yielding a dense and uniform deposition layer, and enhances the interaction between the polymer matrix and α-Fe_2_O_3_ nanomaterials, thereby effectively maintaining structural integrity during fast charge/discharge processes.

**Figure 8:**
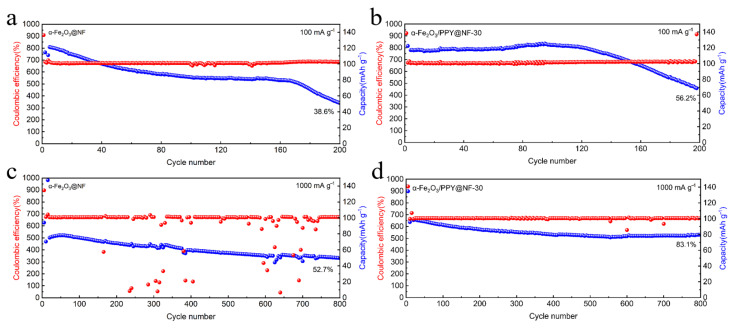
Presents the cycling performance of α-Fe_2_O_3_@NF and α-Fe_2_O_3_/PPY@NF-30 electrodes at current densities of (**a**,**b**) 100 mA g^−1^ and (**c**,**d**) 1000 mA g^−1^.

Figure 8 illustrates the cycling performance and Coulombic efficiency of α-Fe_2_O_3_@NF and α-Fe_2_O_3_/PPY@NF-30 electrodes within a voltage range of 0.01 V to 3.0 V. As depicted in Figure 8a,b, the α-Fe_2_O_3_@NF electrode exhibits a capacity retention of 38.6% after 200 cycles at a current density of 100 mA g^−1^, whereas the α-Fe_2_O_3_/PPY@NF-30 electrode retains 56.2% of its capacity under identical conditions, demonstrating a significant enhancement in cycling stability. The incorporation of PPY effectively mitigates the volumetric expansion of the electrode during lithiation, thereby improving its cycling stability [27,28]. The flexible PPY coating on α-Fe_2_O_3_ buffers the substantial volume changes during lithium insertion/extraction, suppresses particle pulverization, and constructs a continuous electronic transport network, enhancing electrode conductivity. Moreover, the PPY layer isolates the electrolyte from the active material, stabilizes the interface through strong coordination between pyrrole nitrogen and iron ions, and induces the formation of a uniform and robust SEI film, reducing irreversible side reactions and accelerating reaction kinetics. Consequently, the cycling capacity retention of the electrode is substantially improved. Figure 8c,d present the cycling performance and Coulombic efficiency of α-Fe_2_O_3_@NF and α-Fe_2_O_3_/PPY@NF-30 electrodes at a current density of 1000 mA g^−1^ over 800 cycles. Comparative analysis reveals that while the specific capacity decreases under high current conditions, the lifespan is significantly prolonged, particularly for the α-Fe_2_O_3_/PPY@NF-30 electrode. Although α-Fe_2_O_3_@NF exhibits decent performance under high current conditions, its Coulombic efficiency becomes unstable during prolonged cycling [29,30]. The decreased specific capacity under high current conditions is attributed to limited lithium-ion diffusion rates, exacerbated electrode polarization, and insufficient reaction of active materials. However, the rapid charge/discharge process reduces the degree of deep lithiation, and the PPY protective layer stabilizes the structure, isolates electrolyte corrosion, and stabilizes the SEI film, thereby alleviating the volumetric deformation stress of α-Fe_2_O_3_, suppressing particle pulverization and irreversible side reactions, and ultimately extending the electrode’s cycling lifespan.

To directly correlate the enhanced cycling stability with the structural evolution, we examined the morphology and elemental distribution of the electrodes after 100 cycles at 100 mA g^−1^. As shown in Figure S3, the uncoated α-Fe_2_O_3_@NF electrode exhibits severe pulverization, obvious structural collapse, and deep cracks on the electrode surface, which can be attributed to the huge volume variation during repeated lithiation/delithiation. This mechanical degradation inevitably leads to the loss of electrical contact between the active material and the Ni foam current collector, resulting in rapid capacity fading. In sharp contrast, the α-Fe_2_O_3_/PPY@NF-30 electrode (Figure S5) maintains its original nanowire-like morphology and structural integrity without significant agglomeration or cracking. This proves that the flexible PPY shell effectively buffers the internal stress and holds the active material firmly onto the Ni foam scaffold. Furthermore, Figures S4 and S6 display the corresponding EDS-mapping of the cycled electrodes. For the uncoated α-Fe_2_O_3_@NF, the element signals (Ni, Fe, O) become highly aggregated and sparsely distributed due to severe structural fragmentation. However, for the α-Fe_2_O_3_/PPY@NF-30 electrode, the signals of Ni, Fe, O, and N remain uniformly dispersed across the entire selected region. Importantly, the continuous presence of the N signal confirms that the PPY coating layer remains undamaged and tightly attached to the active material even after long-term cycling. This robust and intact PPY coating acts as a physical barrier, isolating the electrolyte from direct contact with the active material, suppressing unwanted side reactions, and promoting the formation of a stable and uniform SEI film. Therefore, the addition of PPY effectively reduces the volume expansion during lithiation, preserves the electrode structural integrity, and significantly enhances the long-term cycling stability.

## 4. Conclusions

This study addresses the pressing demand for high energy density anodes in lithium-ion batteries, focusing on α-Fe_2_O_3_, which exhibits poor conductivity and significant volumetric expansion during charge and discharge cycles. Through a one-step hydrothermal method, α-Fe_2_O_3_ nanowires were in situ grown on a three-dimensional nickel foam scaffold, followed by the electrochemical polymerization of nitrogen-doped polypyrrole (PPY) to achieve a homogeneous coating. This successfully constructed a tri-layer synergistic structure of α-Fe_2_O_3_/PPY@NF-30 as a flexible anode. The results demonstrate that the highly conductive and porous framework of nickel foam ensures rapid electron transport and structural support, while the polypyrrole shell effectively mitigates volumetric effects during charge and discharge processes. Moreover, the in situ nitrogen doping introduces additional active sites, optimizes interfacial compatibility, and facilitates the formation of a stable solid electrolyte interphase (SEI). This composite system achieves a synergistic enhancement of electronic conduction, structural stability, and interfacial reactions, overcoming the performance limitations associated with pure α-Fe_2_O_3_ and single-component composite systems. Notably, the proposed hydrothermal-electropolymerization strategy is highly versatile and can be readily extended to other transition metal oxides (e.g., Co_3_O_4_, MnO_2_, NiO) for advanced alkali-ion batteries. To further improve the reversible capacity and cycle life for practical applications, future efforts could focus on tuning the PPY coating thickness, introducing heteroatom co-doping, or constructing ternary composites with conductive carbon additives. This work provides a straightforward and efficient strategy for the design and fabrication of high-performance lithium-ion battery anodes and offers experimental evidence and theoretical insights for the development of three-dimensional metal substrates/transition metal oxide/conductive polymer composite energy storage materials, demonstrating promising applications in wearable electronics and flexible energy storage devices. Furthermore, evaluating the electrochemical properties of this electrode in a practical full-cell configuration will be a crucial next step toward its commercial application.

## Data Availability

The original contributions pre-sented in this study are included in the article/Supplementary Materials. Further inquiries can be directed to the cor-responding authors.

## Supplementary Materials

The following supporting information can be downloaded at https://www.mdpi.com/article/10.3390/polym18151875/s1.
