# Supplementary material for: Interface Cooperative Enhancement of PPY/Fe2O3@NF Composite Lithium Storage Material
Source: Polymers (Basel). 2026 Jul 30;18(15):1875. doi: 10.3390/polym18151875 (PMC13468665; doi:10.3390/polym18151875)
Supplement: Supplementary file 1 [file polymers-18-01875-s001.zip › polymers-4439532-supplementary.pdf]

Interface Cooperative Enhancement of PPY/Fe_2O_3@NF Composite Lithium Storage Material

Lijun Zhang, Guojing Li, Xiaozhong Qi, Handi Xu, Huaqi Zhao * and Meili Qi *

School of Materials Science and Engineering, Jiamusi University, Jiamusi 154007, China

* Correspondence: zhaohuaqi@jmsu.edu.cn (H.Z.); meili8585@foxmail.com (M.Q.)

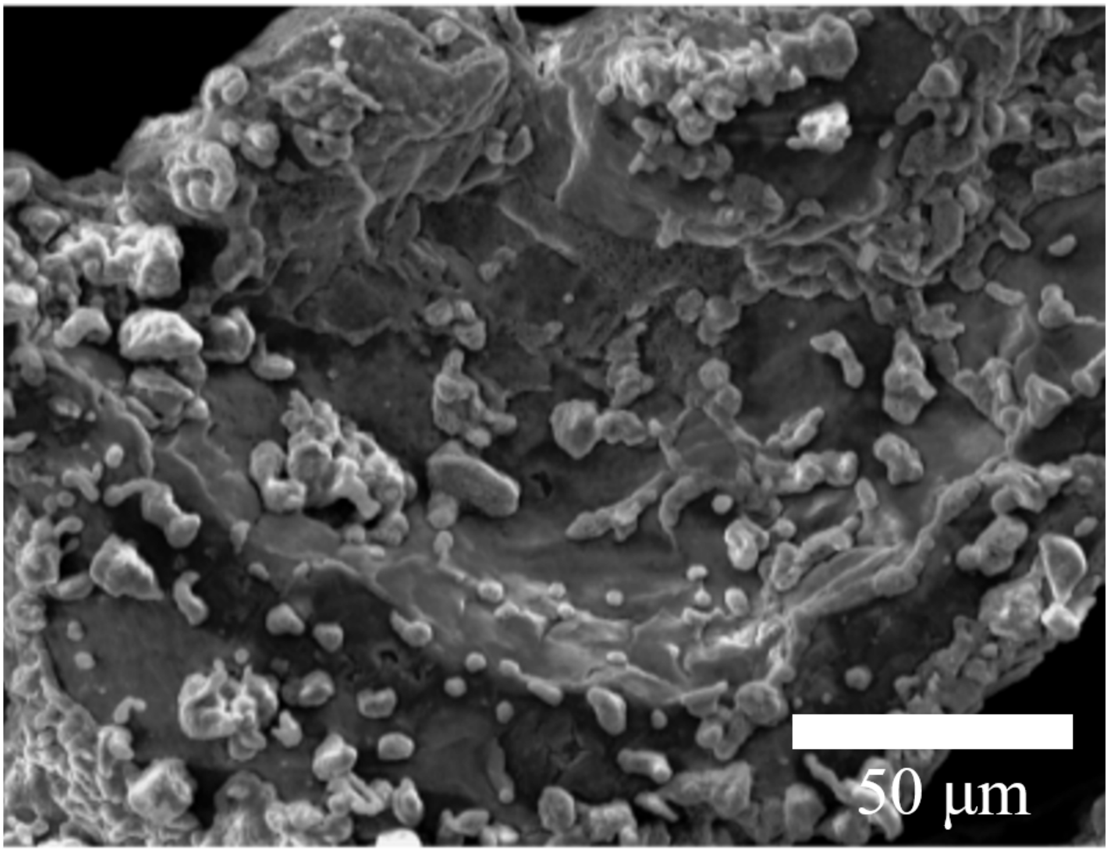

Figure S1. The SEMge of α-Fe_2O_3@NF for EDS.

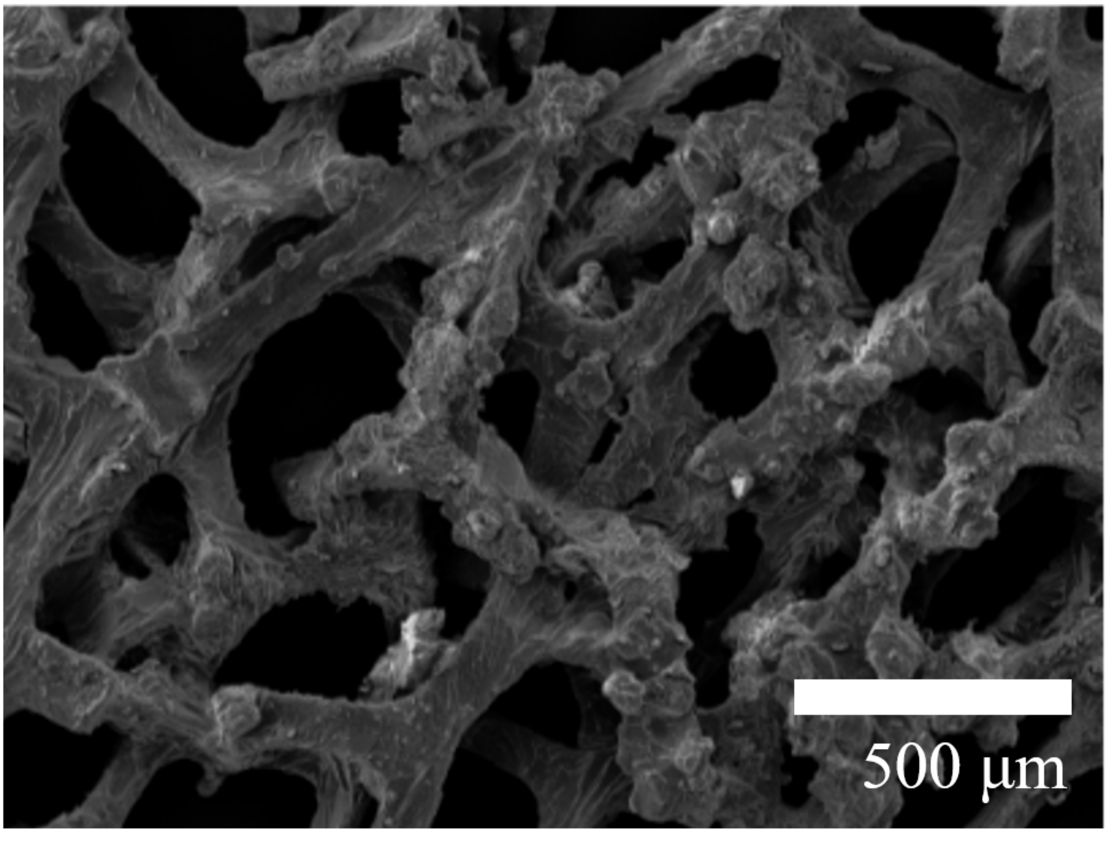

Figure S2. The SEMge of α-Fe_2O_3/PPY@NF-30@NF for EDS.

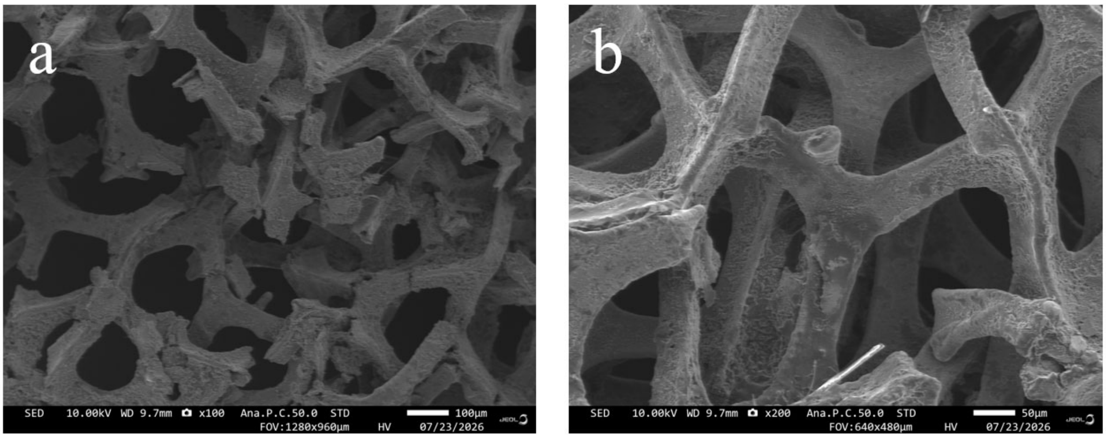

Figure S3. presents SEM micrographs of the α-Fe_2O_3@NF electrode after 100 cycles at a current density of 100 mA g^{-1}.

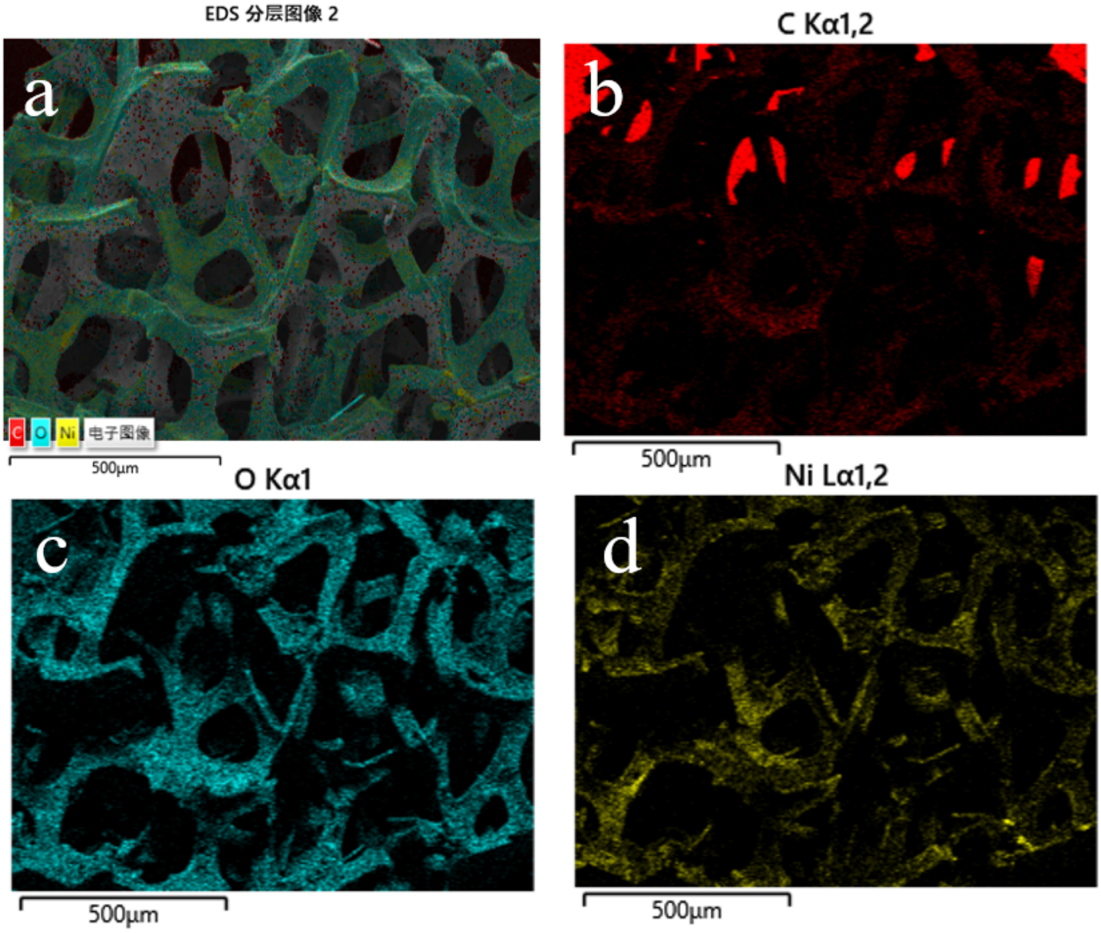

Figure S4. presents EDS-mapping of the α-Fe_2O_3@NF electrode after 100 cycles at a current density of 100 mA g^{-1}.

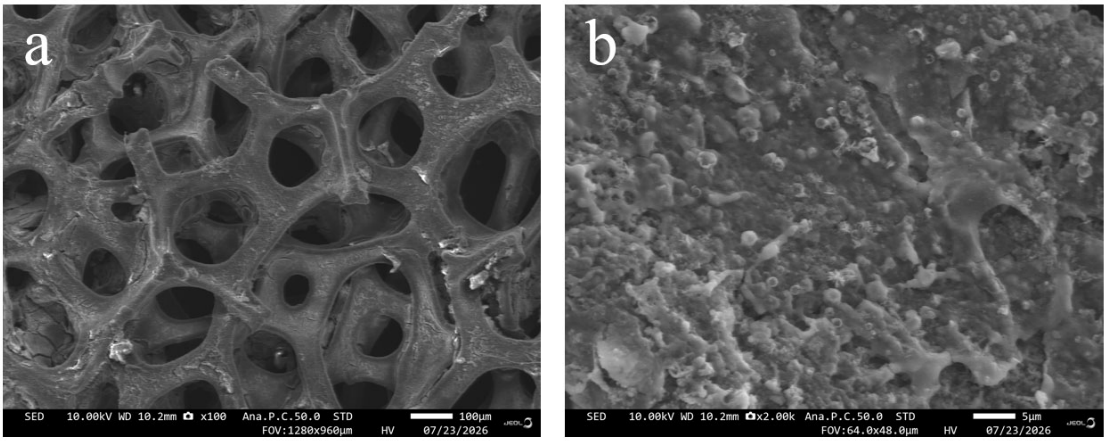

Figure S5. presents SEM micrographs of the α-Fe_2O_3/PPY@NF-30 electrode after 100 cycles at a current density of 100 mA g^{-1}.

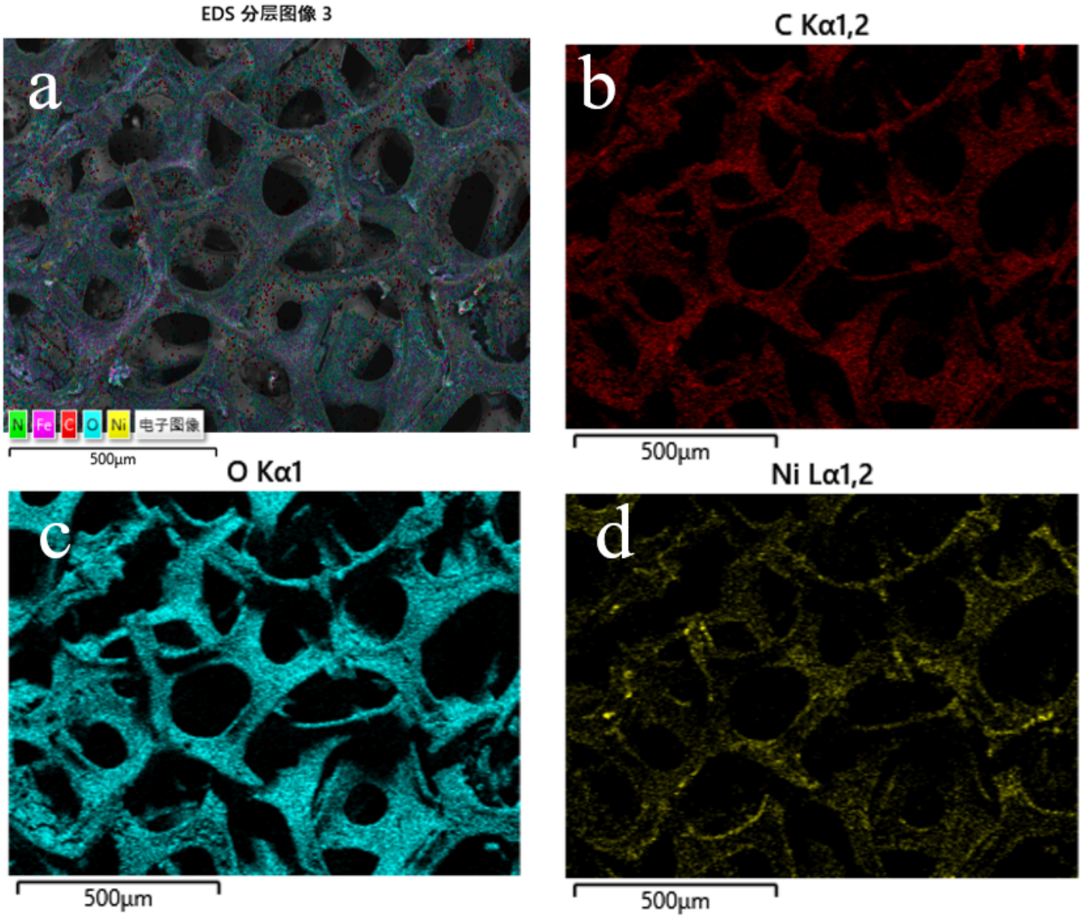

Figure S6. presents EDS-mapping of the α-Fe_2O_3/PPY@NF-30 electrode after 100 cycles at a current density of 100 mA g^{-1}.

Table S1. Electrochemical performance of α-Fe_2O_3/PPY@NF electrodes as a function of deposition time at a current density of 0.1 A g^{-1}.

Deposition Time (s)	Discharge Specific Capacity（mAh/g）	Cycle Life (cycles)	Cycle Retention Rate（%）
0	598	1600	73
15	626	1670	83
30	721	2130	91
45	661	2400	96
60	557	2210	94
